# Carnitine/organic cation transporter 1 precipitates the progression of interstitial fibrosis through oxidative stress in diabetic nephropathy in mice

**DOI:** 10.1038/s41598-021-88724-4

**Published:** 2021-04-27

**Authors:** Shohei Makiishi, Kengo Furuichi, Yuta Yamamura, Keisuke Sako, Yasuyuki Shinozaki, Tadashi Toyama, Shinji Kitajima, Yasunori Iwata, Norihiko Sakai, Miho Shimizu, Tomoko Hirose-Sugiura, Shuichi Kaneko, Yukio Kato, Takashi Wada

**Affiliations:** 1 Department of Nephrology and Laboratory Medicine, Faculty of Medicine, Institute of Medical, Pharmaceutical and Health Sciences, Kanazawa University, Kanazawa, Japan; 2 Department of Nephrology, Kanazawa Medical University School of Medicine, Uchinada, Ishikawa Japan; 3 Faculty of Pharmacy, Institute of Medical, Pharmaceutical and Health Science, Kanazawa University, Kanazawa, Japan; 4 Department of System Biology, Kanazawa University, Kanazawa, Japan

**Keywords:** Nephrology, Kidney diseases

## Abstract

Carnitine/organic cation transporter 1 (OCTN1) is the only known uptake transporter for ergothioneine which is a food-derived strong antioxidant amino acid that is absorbed by OCTN1. We previously reported the roles of OCTN1/ergothioneine in the progression of kidney fibrosis in ischemic kidney disease. In this study, we evaluated the roles of OCTN1 in the progression of diabetic kidney disease. A diabetic kidney disease model was induced in *octn1* knockout and wild-type mice by streptozotocin (STZ). Oxidative stress, represented by urinary 8-hydroxy-2′-deoxyguanosine (8-OHdG), were higher in the *octn1* knockout mice. Azan- and Sirius red-positive areas increased significantly in the *octn1* knockout mice. Gene expression was evaluated by cluster analysis, and shown to be different in the *octn1* knockout mice compared with the wild-type mice. In a pathway analysis, the pathway associated with the cytoskeleton and cell adhesion increased. In accordance with interstitial fibrosis in *octn1* knockout mice, gene expression of moesin in the injured kidney, known as an associated protein of cytoskeleton and cell membranes, was doubled 28 weeks after STZ injection. In addition, the moesin protein was expressed in a part of α-SMA-positive renal tubular epithelial cells. These findings were confirmed by cultured murine proximal tubular epithelial cells: The expression of moesin was induced under oxidative stress with hydrogen peroxide. These data indicate that OCTN1 would play some roles in progression of interstitial fibrosis under oxidative stress via moesin expression in diabetic kidney disease.

## Introduction

Diabetic kidney disease (DKD) is one of the main causes of kidney failure all over the world. Therefore, preventing the progression of DKD is an important social, medical, and economic issue. Among the pathologic features of DKD, specific glomerular lesions, including nodular lesions and mesangiolysis, have been highlighted. However, it is well known that an advanced interstitial lesion is a good prognostic marker in the progression of kidney dysfunction, and this is detectable even in the early stages of DKD^1^. We have previously reported that interstitial fibrosis is an independent, strong prognostic factor for kidney dysfunction using human kidney biopsy samples^2^.

On the other hand, the corticomedullary junction in the kidney is hypoxic, even in a physiological state. This hypoxic condition is obviously enhanced in diabetes^3–5^. Hypoxia is closely associated with oxidative stress. It is speculated that, in the kidney, these may play some part in the progression of interstitial fibrosis. So far, the mechanisms of hypoxia and oxidative stress in the kidney are considered to be mediated by hypoxia-inducible factor (HIF)-regulated erythropoietin, vascular growth factor, and anaerobic glycolysis^6, 7^. However, one specific antioxidative molecule has had little investigation.

Ergothioneine is a food-derived antioxidant amino acid that is transported by carnitin/organic cation transporter 1 (OCTN1)^8^. It is synthesized by mushrooms and some bacteria, in a process that cannot be perfomed by plants or animals^9^. Mammals can absorb ergothioneine through the intestinal tract via OCTN1, which is expressed in various organs including the kidney, liver, and small intestine^8, 10, 11^. The antioxidative and antiinflammatory effects of ergothioneine and OCTN1 have been reported in the small intestine, brain, and kidney^12, 13^. *Octn1* KO mice demonstrated no difference from wild-type (WT) mice under normal conditions. They showed normal growth and no abnormal finding in any organ, including the kidney. Most of the biochemical parameters of the examined sera exhibited a minimal difference between the WT and *octn1* kockout (KO) mice^14^. Moreover, a metabolome study revealed that only ergothioneine was found to be completely absent in the erythrocytes of *octn1* KO mice^14^. Furthermore, we previously evaluated the expression and effect of OCTN1 in the CKD models^13^. However, the role of ergothioneine in diabetic nephropathy has not been evaluated.

Therefore, in this study, we hypothesize that OCTN1 with ergothioneine reduces oxidative stress and suppresses the progression of interstitial fibrosis in DKD.

## Materials and methods

### Mouse model of diabetes

Six-to-eight-week-old male, *octn1* knockout (KO) mice and wild-type (WT) mice with C57BL6 and 129/SvEv F1 backgrounds, were used in a streptozotocin- (STZ, Sigma-Aldrich, St. Louis, MO, USA) induced type 1 diabetes model. STZ was dissolved in sodium citrate buffer (pH 4.5) to a final concentration of 5 mg/ml; this was used as a vehicle. A 5 mg/ml STZ solution was intraperitoneally administered at a dose of 0.05 mg/g mouse body weight. STZ was continuously administered for 4 days at the same dose. After STZ administration, blood glucose and body weight were measured, and urine was collected using a metabolic cage. The animals were sacrificed for histological examination of the kidney at 12 weeks (WT, n = 4; *octn1* KO, n = 4), 20 weeks (WT, n = 11; *octn1* KO, n = 13), and 28 weeks (WT, n = 11; *octn1* KO, n = 12) after STZ administration, at 28 weeks after vehicle(citrate buffer) injection (WT, n = 8; *octn1* KO, n = 8). All procedures used in the animal experiments complied with the standards set out in the guidelines for the care and use of laboratory animals of Kanazawa University, and were approved by the Institute for Experimental Animals, Kanazawa University Advanced Science Research Center (registration number: AP-153390). All animals were also maintained and used in accordance with the ARRIVE guidelines.

### Immunohistochemistry and pathological analysis

The kidney tissue was fixed with formalin and embedded in paraffin. The kidney specimens were stained with Azan, periodic acid Schiff (PAS), and Sirius red (Polysciences, Inc., Warrington, PA, USA). F4/80, CD3, moesin, and α-smooth muscle actin (SMA) were detected using immunohistochemistry. Interstitial fibrosis was evaluated at the corticomedullary junction area by Azan- and Sirius red-stained specimens. Pictures randomly taken around the area were evaluated using image analysis software. Regarding the quantification of histochemical data, we used WinROOF software (Mitani Corporation, Fukui, Japan) to evaluate the area of the stained lesion. The software captured stained lesions on the samples, and the total area was automatically calculated by WinROOF.With respect to the numbers of mice, each group included five mice. Moreover, each of the five randomly chosen fields was quantified using the image analysis software WinROOF. Quantified areas were compared using an unpaired t-test. Moreover, glomerular lesions were evaluated by PAS staining. For immunohistochemistry, deparaffinized sections were treated with proteinase K (Dako, Glostrup, Denmark), peroxidase blocking solution (Dako), and protein block serum-free (Dako). To detect F4/80-positive cells, rat anti-mouse F4/80 Abs (AbD Serotec, Oxford, UK) was used as the primary antibody, and Histofine Simple Stain Mouse MAX-PO (Rat) (Nichirei Bioscience, Tokyo, Japan) was used as the secondary antibody. To detect CD3-positive cells, rabbit anti-CD3 Abs (Dako) was used as the primary antibody, and HRP-EnVision Systems anti-rabbit (Dako) was used as the secondary antibody. The color was developed using diaminobenzidine (DAB) (Dako) and hematoxylin was used as counterstain. α-SMA and moesin were detected by immunofluorescent staining. Deparaffinized sections were treated with proteinase K (Dako), and nonspecific binding was blocked with protein block serum-free (Dako). To detect α-SMA- , aquaporin (AQP1)-, and moesin-positive cells, monoclonal anti-α-smooth muscle actin Ab (clone 1A4, Dako), anti-aquaporin Ab (ab9566, Abcam, Cambridge, UK), and anti-moesin Ab (Abcam) were used as the primary antibody, and Texas Red-conjugated mouse IgG (CAPPEL Organon Teknika Corp., West Chester, PA) and fluorescein-conjugated goat anti-rabbit IgG (CAPPEL Laboratories Cochranville, PA. USA) were used as the secondary antibody. For nuclear staining, 4′, 6-diamidino-2-phenylindole dihydrochloride (DAPI) (Invitrogen) was used.

### Gene expression analysis by real-time PCR

RNA was extracted from mouse kidney tissue using GenElute Mammalian Total RNA Miniprep Kit (Sigma-Aldrich) and converted into cDNA using High-Capacity RNA-to-cDNA Kit (Applied Biosystems, CA, USA). For real-time PCR, iQ SYBR Green Supermix (BIO RAD, CA, USA) was used. The 40-cycle PCR protocol was used at 95 °C for 15 s and 60 °C for 1 min. The data were analyzed using the delta-delta Ct method. The primer for moesin was ATCCGGCAGGGCAACA forward and TGGACGCCCACTACATGGA reverse. For ezrin, it was AGCTCGGAGCGGCTGAT forward and CACTGGTCCCTGCTGAGCTT reverse.

### Measurement of albumin/creatinine (ALB/CRE) and 8-hydroxy-2′-deoxyguanosine (8-OHdG) in urine, and hydroxyproline in kidney tissue

Urinary levels of ALB/CRE and 8-OHdG in the collected samples were evaluated. The urine was immediately centrifuged, and the supernatant was stored at − 30 °C until measurement. Urinary albumin levels were measured using an immune-nephelometric method (LBIS Mouse urinary Albumin Assay Kit, FUJIFILM Wako Shibayagi Co., Shibukawa, Japan), and urinary levels of creatinine levels were measured using an enzymatic method (L-type Wako CRE, FUJIFILM Wako Pure Chemical Co., Osaka, Japan) . New 8-OHdG Check (Nikken Seiru Co., Ltd. Japan Aging Control Research Institute) was used for the measurement of 8-OHdG in urine.

Moreover, the amount of hydroxyproline in mouse kidney tissue was measured using Hydroxyproline Assay Kit (Bio Vision, CA, USA). The kidney tissue was frozen and stored at − 30 °C until measurement.

### Comprehensive mRNA expression analysis using a next-generation sequencer

The serial analysis of gene expression (SAGE) method was performed using Ion Torrent PGM (Life technologies). RNA expression in four groups of *octn1* KO and WT mice with STZ or control were evaluated. RNA from the kidneys of the animals in each group was mixed in equal amounts and used for analysis. A SAGE library was prepared from 1 μg of RNA extracted from mouse kidney tissue, according to the manufacturer’s instructions for the SOLID SAGE Kit (Life technologies). The sample obtained was analyzed by Ion Torrent PGM using 318 chips. The data obtained from the sequencer was analyzed with MetaCore.

### In vitro study

The expression of moesin was confirmed using the tubular epithelial cell line mProx24. The cells were cultured in DMEM containing 10% heat-inactivated FBS, 100 U/ml penicillin, and 100 μg/ml streptomycin. Cells (2 × 10^6^ /well) were incubated in a 24-well tissue culture plate for 24 h, then made quiescent by incubation with 0.1% FBS-DMEM for 24 h. Quiescent cells were incubated with or without high concentration D- or L-glucose, or H_2_O_2_ for 6, 12, and 24 h. Some part of high D-glucose stimulation sets was also cultured with ergothioneine. RNA was extracted from the cells in the same way as from the kidney samples, and gene expression was evaluated by real-time PCR.

### Statistical analysis

Data are expressed as mean ± standard deviation. Statistical analyzes were performed using an unpaired Student’s t-test, Kruskal–Wallis test, and analysis of variance. All analyzes were conducted using Stata version 13 (StataCorp, LLC; College Station, TX, USA). A 2-sided P value of < 0.05 was considered statistically significant.

## Results

### Glomerular lesion in STZ-induced diabetic mouse

**Figure 1:**
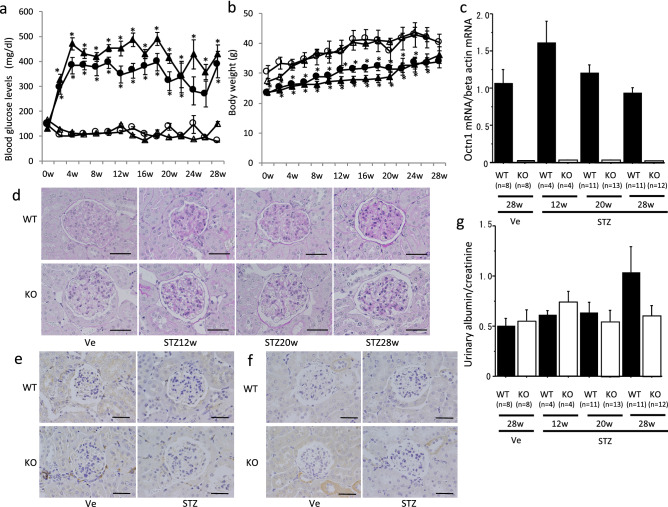
Glomerular lesion in STZ-induced diabetic mice. STZ or Vehicle control (Ve) solution was injected into the intraperitoneal cavity of 6–8 week-old male *octn1* KO and WT mice. Blood glucose levels (**a**) and body weight (**b**) of the *octn1* KO and WT mice (circle: WT-Ve, triangle: KO-Ve, filled circle: WT-STZ, filled triangle: KO-STZ) were shown. OCTN1 mRNA expression was only detected in the WT mice (**c**). Glomerular images by PAS staining were shown in panel (**d**) before and after STZ injection. F4/80-positive (**e**) and CD3-positive cells (**f**) were faintly detected in the glomeruli. Urinary levels of albumin in the *octn1* KO and WT mice was almost no increase throughout the observation period (**g**). KO, *octn1* KO mice; WT, wild-type mice; STZ, STZ injected mice; Ve, vehicle control.*P < 0.05 for each control. The scale bar represents 100 μm.

Blood glucose apparently increased in the STZ-induced diabetic mice from the second week after STZ administration, and reached 400–500 mg/dL at the fourth week. The increased blood glucose levels in the STZ-induced animals were not significantly different between *octn1* KO and WT mice, although the levels in *octn1* KO mice tended to be slightly higher than WT mice (Fig. 1a). Body weight was lower in the diabetic group than in the control group. There was no difference in body weight between the *octn1* KO and WT mice (Fig. 1b). As shown in Fig. 1a,b, four groups were evaluated in this sudy: nondiabetic WT, diabetic WT, nondiabetic *octn1* KO and diabetic *octn1* KO 28 weeks after STZ. We also checked pathological changes at 28 weeks after vehicle (citrate buffer) injection, both in the WT and *octn1* KO mice. The pathological findings were normal, with no difference between 0-week and 28-week animals in the vehicle group both in the WT type and *octn1* KO mice. Therefore, we mainly evaluated the following study in diabetic WT and *octn1* KO mice. The expression level of OCTN1 mRNA in the kidneys of the WT mice did not change after STZ administration (Fig. 1c). Pathological evaluation under the microscope with PAS staining showed mild glomerular hypertrophy in the kidneys of both *octn1* KO and WT diabetic mice. However, neither nodular nor diffuse lesions were detected, even in the diabetic mice at 28 weeks after STZ administration (Fig. 1d). In addition, F4/80-positive cells and CD3-positive cells were faintly observed in the glomeruli of diabetic mice (Fig. 1e,f). Moreover, albuminuria was almost unchanged in diabetic *octn1* KO and WT mice (Fig. 1g).

### Oxidative stress and interstitial fibrosis were advanced in octn1 KO diabetic mice

**Figure 2:**
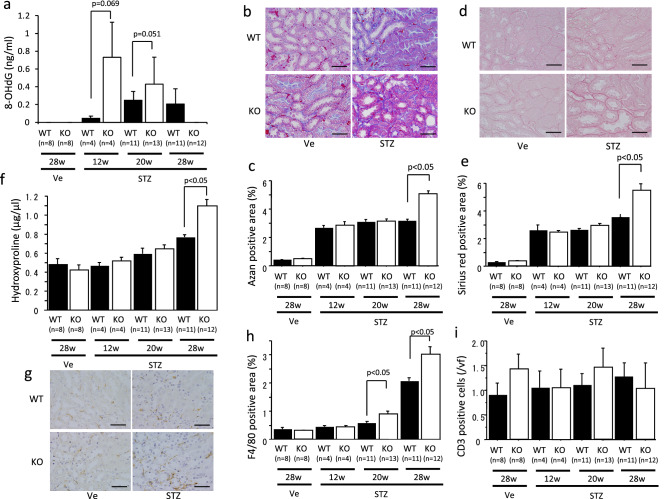
Evaluation of oxidative stress and interstitial fibrosis in STZ-induced diabetic mice. Urinary 8-OHdG levels were detected in the diabetic mice (**a**). Interstitial fibrosis was detected by Azan staining (**b**) and Sirus red staining (**d**), and the fibrotic areas were quantified (**c**,**e**). The amount of hydroxyproline in the mice kidneys was measured (**f**). Interstitial F4/80-positive cells were detected by immunohistochemistry (**g**) and their positive area was evaluated (**h**). The number of CD3-positive cells was also detected by immunohistochemistry and counted (**i**). KO, *octn1* KO mice; WT, wild type mice; STZ, STZ injected mice; Ve; vehicle control. The scale bar represents 100 μm.

Oxidative stress in the kidneys was evaluated by detecting urinary 8-OHdG. Levels of this were undetectable in the normal mice, but significantly increased in the WT mice after STZ injection. The increased urinary 8-OHdG levels at the 12th and 20th weeks after STZ injection were higher in the *octn1* KO mice than in the WT mice (Fig. 2a). Moreover, the Azan-positive area increased after STZ injection and was significantly wider in the *octn1* KO mice than in the WT mice at 28 weeks (Fig. 2b,c). Similarly, the Sirius red-positive area increased after the induction of diabetes and was significantly wider in the *octn1* KO mice than in the WT mice at 28 weeks (Fig. 2d,e). To confirm these histological findings, the amount of hydroxyproline, a biochemical method of quantifying collagen, was evaluated. The amount of hydroxyproline gradually increased after the induction of diabetes, and the content of hydroxyproline increased significantly in the *octn1* KO mice, compared to the WT mice, at 28 weeks after STZ injection (Fig. 2f). Furthermore, interstitial inflammatory cell infiltration was evaluated by immunohistochemistry. The F4/80-positive area gradually expanded after diabetes induction. At weeks 20 and 28, the F4/80-positive area in the *octn1* KO mice was larger than that of the WT mice (Fig. 2g,h). However, the number of CD3-positive cells in the interstitial did not increase, even after the induction of diabetes, and there was no difference between the *octn1* KO and WT mice (Fig. 2i).

### The cytoskeleton- and cell adhesion-related pathways were upregulated in octn1 KO diabetic mice

**Figure 3:**
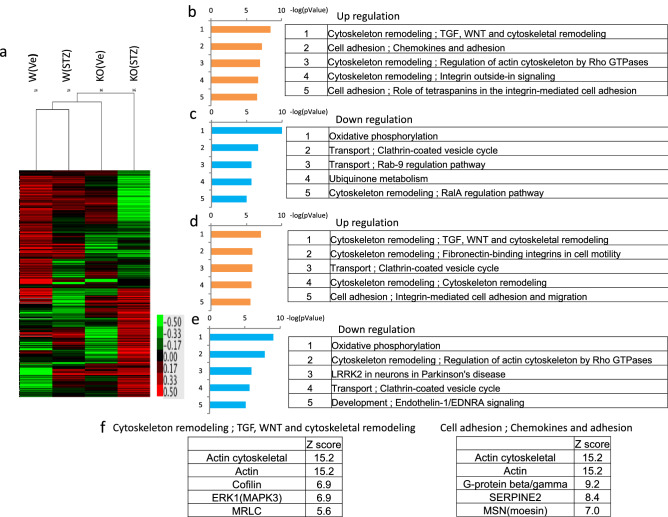
Comprehensive gene analysis in the kidney, and cluster and pathway analysis. As a result of the cluster analysis, gene expression of the 4 groups (WT-Ve, KO-Ve, WT-STZ, KO-STZ) was seen to be significantly different (**a**). Upregulated (**b**) and downregulated (**c**) pathways were listed to compare the WT-STZ and KO-STZ groups. Upregulated (**d**) and downregulated (**e**) pathways were listed to compare the WT-Ve and WT-STZ groups. Lists of high Z-scored molecules included in the cytoskeleton- and cell adhesion-related pathways were shown (**f**). KO, *octn1* KO mice; WT, wild type mice; STZ, STZ injected mice; Ve, vehicle control.

Comprehensive gene expression in the STZ-induced diabetic mice at 28 weeks were analyzed by Ion Torrent. Using gene expression data, cluster and pathway analyses were carried out by MetaCore. Cluster analysis revealed that gene expression patterns before diabetes were different between the *octn1* KO and WT mice (Fig. 3a). In addition, the gene expression pattern after STZ injection was also different between the two groups. Moreover, pathway analysis revealed that cytoskeleton- and cell adhesion-related pathways were upregulated in the diabetic *octn1* KO mice, compared to the diabetic WT mice (Fig. 3b). In contrast, oxidative phosphorylation- and intracellular transport-related pathways were downregulated in the diabetic *octn1* KO mice, compared to the diabetic WT mice (Fig. 3c). Compared before and after the induction of diabetes in the WT mice, cytoskeleton- and cell adhesion-related pathways were upregulated (Fig. 3d) and oxidative phosphorylation-related pathways were downregulated (Fig. 3e). Lists of high Z-scored molecules included in the cytoskeleton- and cell adhesion-related pathways were shown (Fig. 3f). Z score of moesin was 7.0 in the cell adhesion-related pathways.

### Moesin expression was upregulated in interstitial α-SMA-positive cells in octn1 KO diabetic mice

**Figure 4:**
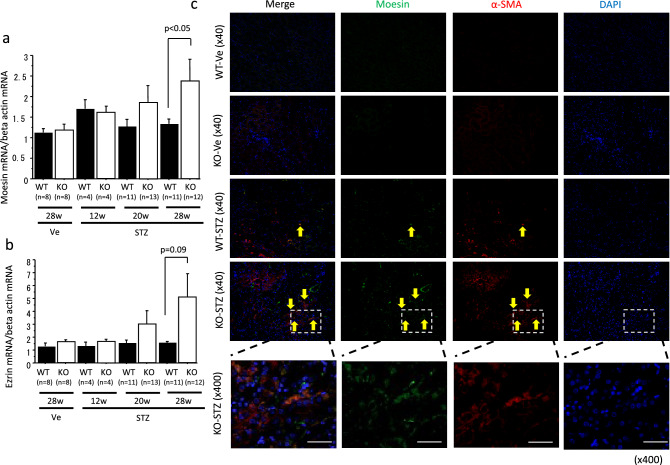
Moesin co-expressed on α-SMA-positive cell. Expression of moesin and ezrin was evaluated in the kidneys of the *octn1* KO and WT mice with/without diabetes (**a**,**b**). Double positive cells of α-SMA and moesin were detected in *octn1* KO diabetic mice .Yellow arrows indicates Moesin, α-SMA, and Moesin and α-SMA dual- positive cells. (**c**) KO, *octn1* KO mice; WT, wild type mice; STZ, STZ injected mice; Ve, vehicle control.. The scale bar represents 100 μm.

Among the molecules in the cytoskeleton and cell adhesion pathways, we focused on moesin. mRNA and protein expression were evaluated in mice kidneys using moesin and its relative, ezrin. Real-time PCR studies revealed that moesin and ezrin were upregulated in the *octn1* KO diabetic mice. The expression level of moesin in these was higher than it was in the WT mice at 28 weeks after STZ injection (Fig. 4a,b). On the other hand, typical fibrosis-related factors, such as TGF-β, CTGF, and PDGF-B, showed no difference between the *octn1* KO and WT groups (data not shown). Immunohistochemical analysis indicted that moesin protein expression overlapped in α-SMA-positive cells in the *octn1* KO mice at 28 weeks after STZ injection. Moreover, moesin positives were also overlapped in AQP1 (a marcher of proximal tubular cell)-positive (Supplementary Fig. 1). However, almost no α-SMA- and moesin-positive cells were observed in the nondiabetic *octn1* KO and WT mice. α-SMA- and moesin-positive cells were tubular epithelial in the *octn1* KO mice at 28 weeks after STZ injection (Fig. 4c).

### Moesin expression was confirmed under the oxidative stress in in vitro study

**Figure 5:**
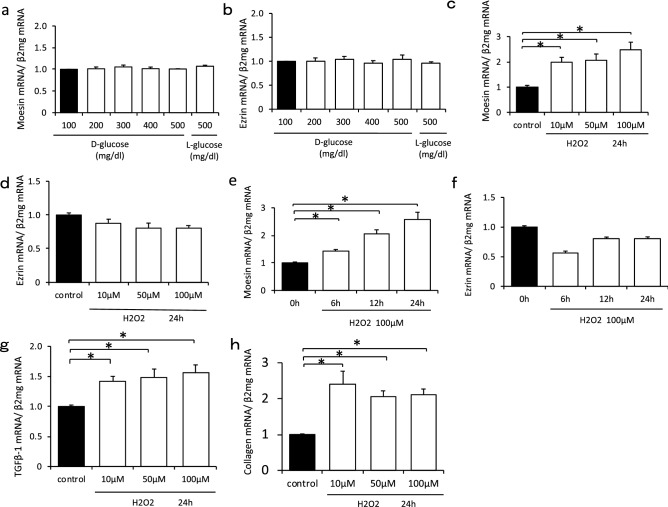
In vitro study for moesin expression under conditions of high glucose or oxidative stress. Moesin and ezrin mRNA expression did not change under the high glucose condition (**a**,**b**). Moesin mRNA expression was upregulated under hydrogen peroxide stimulation (**c**). Moesin mRNA expression increased in a time-dependent manner under the condition of 100 µM hydrogen peroxide stimulation (**e**). Ezrin mRNA expression did not change (**d**,**f**). The expression of Tgfb1 was enhanced under the condition of hydrogen peroxide stimulation (**g**). The expression of collagen 1 was also upregulated in the same condition (**h**).

Moesin and ezrin mRNA expression did not change under the high glucose conditions (Fig. 5a,b). However, moesin mRNA expression was upregulated under hydrogen peroxide stimulation (Fig. 5c). Moreover, moesin mRNA expression increased in a time-dependent manner under the condition of 100 µM hydrogen peroxide stimulation (Fig. 5e). However, ezrin mRNA expression did not change (Fig. 5d,f). Furthermore, the expression of Tgfb1 was enhanced, under the condition of hydrogen peroxide stimulation, and the expression of collagen 1 was also upregulated in the same condition (Fig. 5g,h). Moreover, high glucose stimulation induced Tgfb1 was reduced by ergothioneine treatment (Supplementary Fig. 2).

## Discussion

In this study, we revealed that oxidative stress, represented by urinary 8-OHdG, was higher in the *octn1* KO mice than in the WT mice. Moreover, interstitial fibrosis progressed more in the diabetic *octn1* KO mice than that the diabetic WT mice. In addition, moesin protein was expressed in α-SMA-positive renal tubular epithelial cells, and had a part in interstitial fibrosis.

In this study, oxidative stress was stronger in the *octn1* KO mice, in which interstitial fibrosis progressed. In diabetic nephropathy, interstitial fibrosis is an important pathological change related to kidney dysfunction. The kidney has a strong hypoxic condition and oxidative stress in normal conditions, and the hypoxic condition and oxidative stress are further enhanced in the diabetic state^3–5^. In this study, we focused on OCTN1, the only known transporter for ergothioneine, which has strong antioxidant activity. Since ergothioneine is absorbed by the intestine via OCTN1, *octn1* KO mice had no ergothioneine in their bodies at all^14^. The oxidative stress shown by 8-OHdG increased in the diabetic model mice from the 12th week after STZ injection. Urinary 8-OHdG, which shows oxidative stress, was high at 12 and 20 weeks in the STZ-induced diabetes model, while histological difference in fibrosis was observed at 28 weeks. To explain the time lag between oxidative stress and fibrosis, collagen production may have been accumulating under oxidative stress, and it took a longer period to show a histological phenotype. In addition, it is speculated that inflammatory processes may have had some role in fibrosis under the oxidative stress of the diabetic mice. Actually, our data showed that F4/80-positive cells had increased in the *octn1* KO diabetic mice. Moreover, a pathway analysis showed that G protein-related molecule expression had been enhanced downstream of the chemokine/cytokine receptor upregulated in the *octn1* KO diabetic mice. We previously reported a relationship between inflammatory cells, including F4/80-positive cells and kidney interstitial fibrosis^15–17^. Such fibrosis also requires time for inflammatory cell infiltration, and this would be an additional reason why the oxidative stress and the actual fibrosis had a time lag. Urinary 8-OHdG level in *octn1* KO mice at 28 weeks were very low in this study. However, fibrosis was progressed by the 28 weeks following STZ injection in the *octn1* KO mice. Urinary 8-OHdG levels were gradually increased in WT mice until 28 weeks after STZ injection. Urinary 8-OHdG levels in *octn1* KO mice were significantly higher than those of WT mice at 12 and 20 weeks after STZ injection. However, 8-OHdG levels peaked at 12 weeks after STZ injection and decreased thereafter in *octn1* KO mice. We speculate that oxidative stress was induced in the early phase (12–20 weeks) of the diabetic condition in *octn1* KO mice due to ergothioneine deficiency. During this period, fibrogenic processes were induced and collagen deposition was accumulated until 28 weeks after STZ injection. In contrast to these fibrogenic processes, some antioxidative processes, such as Keap1-Nrf2 induced gene expression, may be activated and may subsequently reduce oxidative stress in *octn1* KO mice. According to the genetic background of OCTN1 deficiency may accelerate the induction of antioxidative processes.

Therefore, it is speculated that oxidative stress in *octn1* KO diabetic mice had induced interstitial fibrosis through direct and indirect mechanisms.

Our data showed that moesin expression was enhanced along with interstitial fibrosis. Moesin is a part of the ERM family, which also contains ezrin and radixin. Moesin connects transmembrane proteins with the actin cytoskeleton, and maintains the polarity of epithelial cells^18, 19^. In addition to these functions, it was recently reported that moesin also had a part in extracellular signal transduction^20^. Moreover, it has been reported that cerebral cavernous malformation 3 (CCM3), a binding molecule of moesin, plays some part in intracellular survival signals under the oxidative stress. This moesin-related signaling pathway is associated with fibrosis^21, 22^. Moreover, moesin is reported to be co-expressed with α-SMA and involved in corneal fibrosis via TGF-β^23^. Furthermore, the association of moesin in liver fibrosis has also been reported using moesin KO mice^24^. These findings support our observation of the increased expression of moesin, and co-expression with α-SMA, in accordance with the progression of fibrosis under oxidative stress. However, the detailed molecular mechanism of moesin in fibrosis has not been fully clarified, and further studies are required.

In this study, the glomerular lesions and albuminuria were mild, but interstitial fibrosis progressed under oxidative stress. In diabetic nephropathy, the degree of proteinuria is a strong prognostic factor^2^. However, some cases of rapidly decreased kidney function with minor albuminuria have been reported^25, 26^. Therefore, it is important to clarify the mechanisms of interstitial fibrosis. It is difficult, however, to distinguish the effects of albuminuria and other factors of interstitial fibrosis in general because albuminuria affects the progression of interstitial fibrosis through the tubular epithelial cell stress. However, our model showed minor albuminuria and advanced interstitial fibrosis. We believe this model can assist in evaluating interstitial fibrosis without albuminuria. In this study, we administered a small amount of STZ frequently, and could create a model with hyperglycemia and minor glomerular lesions, but not albuminuria. In addition to our protocol of STZ administration, the mouse’s strain may have had some role in this phenotype. It was reported the susceptibility of the STZ model was different among mouse strains^27^. Moreover, STZ injection protocols also show some variation among studies. C57BL6 mice are resistant to glomerular changes following STZ. A previous study revealed that C57BL6 mice receiving an intraperitoneal STZ injection (0.05 mg/g) for five consecutive days did not develop albuminuria or glomerular lesions at 45 weeks^27^. We believe that this finding is consistent with our study.

In conclusion, our data indicate that OCTN1 can have a role in the progression of interstitial fibrosis under oxidative stress via moesin expression in diabetic kidney disease.

## Supplementary information

Supplementary Information 1.

Supplementary Information 2.
