# Supplementary material for: Carnitine/organic cation transporter 1 precipitates the progression of interstitial fibrosis through oxidative stress in diabetic nephropathy in mice
Source: Sci Rep. 2021 Apr 27;11:9093. doi: 10.1038/s41598-021-88724-4 (PMC8079701; doi:10.1038/s41598-021-88724-4)
Supplement: Supplementary file 1 — Supplementary Information 1. [file 41598_2021_88724_MOESM1_ESM.pdf]

Supplementary Figure 1

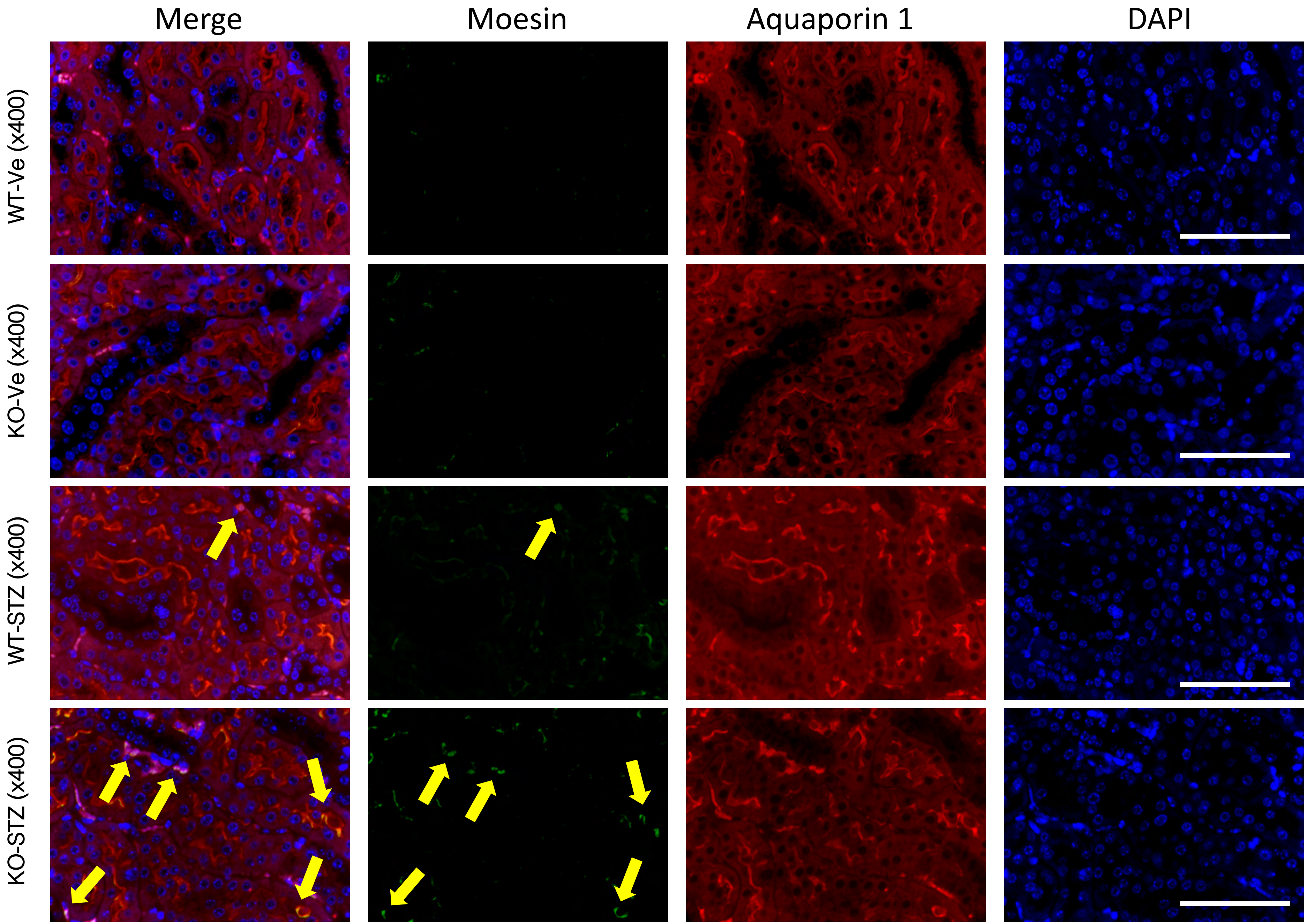

LEGENDS

Supplementary Figure 1

Main part of moesin-positive cells were AQP1-positive proximal tubular epithelial cells.

Expression of moesin was evaluated in the kidneys of the *octn1* KO and WT mice with/without diabetes. Moesin-positive cells were mostly detected in *octn1* KO diabetic mice .Yellow arrows indicates Moesin and AQP1 dual- positive proximal tubular epithelial cells. (c) KO, *octn1* KO mice; WT, wild type mice; STZ, STZ injected mice; Ve, vehicle control. The scale bar represents 100 μm.
