# Supplementary material for: Carnitine/organic cation transporter 1 precipitates the progression of interstitial fibrosis through oxidative stress in diabetic nephropathy in mice
Source: Sci Rep. 2021 Apr 27;11:9093. doi: 10.1038/s41598-021-88724-4 (PMC8079701; doi:10.1038/s41598-021-88724-4)
Supplement: Supplementary file 2 — Supplementary Information 2. [file 41598_2021_88724_MOESM2_ESM.pdf]

Supplementary Figure 2

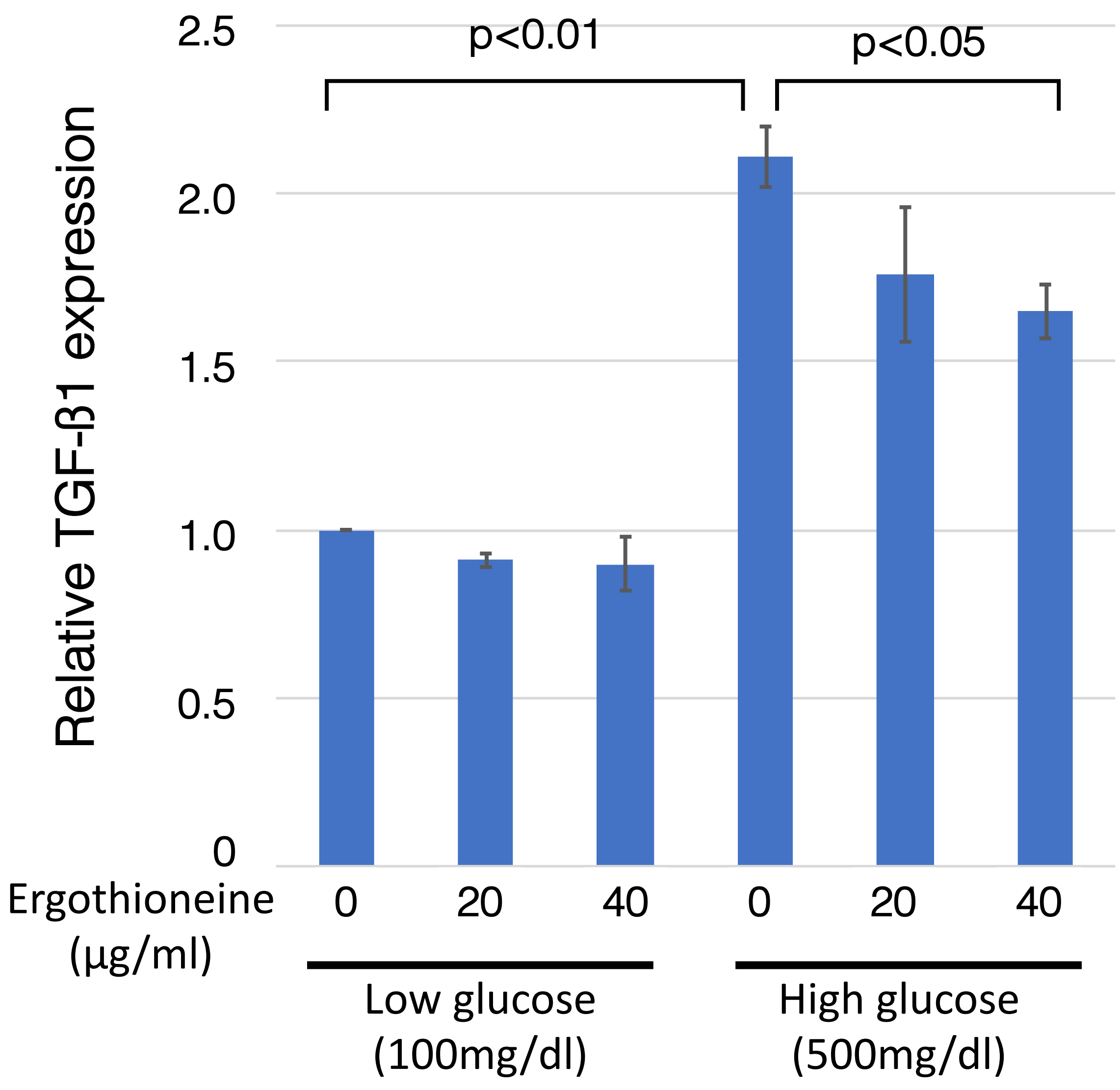

LEGENDS

Supplementary Figure 2

Ergothionein reduced Tgfb1mRNA expression under conditions of high glucose stimulation.

The effects of ergothioneine in expression of Tgfb1 was evaluated using the tubular epithelial cell line. The cells were cultured in DMEM containing 10% heat-inactivated FBS, 100 U/ml penicillin, and 100 μg/ml streptomycin. Cells were incubated for 24 h, then made quiescent by incubation with 0.1% FBS-DMEM for 24 h. Quiescent cells were incubated with or without high concentration D-glucose (500 mg/dl) for 24 h with or without ergothioneine (20, 40 μg/ml). The expression of Tgfb1 was enhanced under the condition of high glucose stimulation, and the expression was reduced by ergothioneine.
